# Retraction: The effect of MCM3AP-AS1/miR-211/KLF5/AGGF1 axis regulating glioblastoma angiogenesis

**DOI:** 10.3389/fnmol.2023.1275841

**Published:** 2023-08-24

**Authors:** Frontiers Editorial Office

The journal retracts the 9 January 2018 article cited above.

Following publication, concerns were raised regarding the integrity of the images in the published figures, with areas of image duplication in Figure 3E. The authors failed to provide a satisfactory explanation during the investigation, which was conducted in accordance with Frontiers' policies. As a result, the data and conclusions of the article have been deemed unreliable and the article has been retracted.

This retraction was approved by the Chief Editors of Frontiers in Molecular Neuroscience and the Chief Executive Editor of Frontiers. The authors did not agree to this retraction.

